# Effect of intraoperative platelet-rich plasma on inferior alveolar nerve neurosensory outcomes after high-risk mandibular third molar extraction

**DOI:** 10.4317/medoral.28126

**Published:** 2026-04-19

**Authors:** Jose Carlos Roman-Padilla, Eduardo Alavedra Salgueiro, Pol Alavedra Martínez, Luis Orti Ortiz-Peces, Diego Sanchez Garnica-Toquero, María Castillo Pereira, Álvaro Sada Malumbres, Javier González Martín-Moro, Jose Luis Cebrian-Carretero

**Affiliations:** 1 La Paz University Hospital, Madrid, Spain. La Paz Hospital Institute for Health Research (IdiPAZ), Madrid, Spain

**Keywords:** Inferior alveolar nerve, platelet-rich plasma, third molar extraction, neurosensory impairment, nerve injury prevention.

## Abstract

**Background:**

Inferior alveolar nerve (IAN) injury is a relevant complication of mandibular third molar extraction, particularly in radiologically high-risk cases. Platelet-rich plasma (PRP) is a safe and accessible autologous product, but its preventive effect on IAN neurosensory impairment remains unknown.

**Materials and Methods:**

An ambispective cohort study included 300 high-risk mandibular third molar extractions. Patients received intraoperative PRP (n=144) or no PRP (n=156). The primary outcome was IAN neurosensory impairment at 1 week. Secondary outcomes were time to recovery and persistence at 6 months. Multivariable and propensity score-weighted analyses were performed.

**Results:**

IAN impairment at 1 week occurred in 18.0% of cases and was less frequent in the PRP group (11.1% vs 24.4%), with an absolute risk reduction of 13.2% (number needed to treat=8). PRP use remained independently associated with lower early IAN impairment. Group differences were evident after 2 months. PRP was not associated with shorter recovery time or reduced long-term persistence. Increasing age was associated with worse neurosensory outcomes.

**Conclusions:**

In a radiologically high-risk population undergoing mandibular third molar extraction, intraoperative platelet-rich plasma use was associated with a reduction in early inferior alveolar nerve neurosensory impairment, without a measurable effect on long-term recovery or persistence. These findings suggest that PRP may act as a preventive intervention during the acute postoperative phase rather than as a treatment for established nerve injury.

## Introduction

Inferior alveolar nerve (IAN) injury is a well-recognized and clinically significant complication following the surgical extraction of mandibular third molars. The estimated incidence of IAN injury ranges from 0.4% to 8.4%, with permanent injury occurring in 0.014% to 3.6% of cases. In high-risk populations, the likelihood of IAN injury has been reported to increase to 10-35% [1,2]. Although most neurosensory disturbances are transient and resolve within the first weeks after surgery, even temporary sensory deficits affecting the lower lip, chin, and adjacent mucosa may lead to functional impairment, altered speech and mastication, and a substantial reduction in quality of life. When sensory alteration persists beyond six months, it is generally considered permanent, with long-term physical, psychological, and social consequences [3]. In addition, iatrogenic injury to the IAN represents one of the most frequent causes of medicolegal claims in oral and maxillofacial surgery, highlighting the importance of prevention rather than delayed treatment once injury has occurred [4].

Mandibular third molar extraction is among the most commonly performed surgical procedures in oral and maxillofacial practice worldwide. In England, for instance, it is estimated that approximately 152,000 people undergo this procedure annually [5]. As a result, even relatively low incidence rates of nerve injury translate into a substantial absolute number of affected patients. The risk of IAN impairment increases substantially in anatomically complex cases, particularly when cone-beam computed tomography (CBCT) demonstrates intimate contact between the mandibular canal and the tooth. In these high-risk scenarios, surgical removal typically requires osteotomy and odontosection, which may increase the likelihood of direct or indirect neural trauma through compression, stretching, ischemia, or inflammatory mechanisms. Despite careful surgical planning and technical refinements, current preventive strategies remain limited, and systematic reviews have failed to identify any surgical approach with robust evidence for reducing IAN injury risk [6].

To date, most strategies addressing IAN injury have focused on postoperative management rather than intraoperative prevention. Surgical repair, pharmacological therapies, low-level laser therapy, and psychological interventions have all been proposed to enhance neurosensory recovery once injury has occurred. However, high-quality evidence supporting these interventions is scarce, with systematic reviews consistently reporting small sample sizes, high risk of bias, and very low certainty of evidence. Importantly, these approaches can only be applied after nerve injury has already been established, inherently limiting their preventive value [4].

In this context, platelet-rich plasma (PRP) has emerged as a biologically plausible adjunct in oral and maxillofacial surgery. PRP is an autologous blood-derived product containing a high concentration of platelets and growth factors involved in angiogenesis, modulation of inflammation, and tissue repair. Its use has been extensively investigated in dental and maxillofacial procedures, particularly for soft tissue healing and bone regeneration, and it is generally regarded as safe, cost-effective, and technically simple to prepare [7]. From a theoretical standpoint, the local application of PRP at the surgical site may influence the neural microenvironment immediately after injury, potentially mitigating secondary neural damage and supporting early recovery. However, these effects remain hypothetical in the specific context of inferior alveolar nerve injury.

Despite the growing body of literature on platelet concentrates in third molar surgery, existing studies have primarily focused on postoperative pain, swelling, trismus, alveolar osteitis, and bone healing. Neurosensory outcomes related to the IAN have been inconsistently reported or entirely omitted. Recent systematic reviews and meta-analyses confirm the heterogeneity of study designs, platelet concentrate protocols, outcome measures, and follow-up periods [8,9]. Notably, no study to date has specifically evaluated the intraoperative use of PRP as a preventive measure for inferior alveolar nerve neurosensory impairment.

Given the high prevalence of mandibular third molar surgery, the potentially disabling nature of IAN injury, and the absence of targeted evidence addressing preventive intraoperative strategies, further investigation is warranted. Real-world data derived from well-defined high-risk populations may help clarify whether PRP merits consideration as a preventive adjunct in selected cases.

The aim of the present study was to evaluate the association between intraoperative application of platelet-rich plasma and the incidence of inferior alveolar nerve neurosensory impairment after surgical extraction of mandibular third molars in a CBCT-defined high-risk population. Secondary objectives were to assess time to neurosensory recovery and the persistence of impairment at six months of follow-up.

## Material and methods

*Ethics statement*

The study was conducted in accordance with the Declaration of Helsinki and approved by the Research Ethics Committee for Medicines (CEIm) of La Paz University Hospital, which issued a favourable opinion for this observational study with medicines (internal code 2025.458; HULP code PI-6754). The study was classified as non-commercial research and conducted in compliance with Royal Decree 957/2020. Patient data were handled in a coded manner in accordance with the General Data Protection Regulation (GDPR) and applicable national legislation.

*Study design*

An ambispective observational cohort study was conducted to evaluate the association between intraoperative platelet-rich plasma (PRP) application and inferior alveolar nerve (IAN) neurosensory impairment following mandibular third molar extraction. All eligible mandibular third molar extractions performed at the Department of Oral and Maxillofacial Surgery from 2021 onward were included. The cohort was identified both retrospectively and prospectively. Postoperative neurosensory follow-up was carried out according to routine clinical practice and documented in the electronic medical records.

A total of 300 mandibular third molar extractions were analysed. The unit of analysis was the tooth, and no patient contributed more than one third molar. Patients were allocated into two cohorts according to PRP exposure: A PRP group (n=144) and a non-PRP group (n=156). Baseline and surgical variables collected included age, gender, tooth side (38 or 48). No patients presented relevant systemic comorbidities or regular medication use that could influence nerve recovery. PRP use reflected routine clinical practice during the study period. Its application depended mainly on logistical availability of preparation materials during the surgical session and on the surgeon’s clinical judgment in cases perceived as potentially more complex or technically demanding. These factors explain the coexistence of PRP and non-PRP cases within the cohort and may partially account for the higher mean age observed in the PRP group.

Inclusion criteria comprised: Age ≥18 years, surgical extraction of a mandibular third molar, availability of preoperative cone-beam computed tomography (CBCT), and a high-risk radiological relationship between the mandibular third molar and the inferior alveolar nerve, defined as a >30% opening of the mandibular canal toward the alveolus on multiplanar CBCT reconstructions. All CBCT scans were reviewed by a single trained evaluator applying the same predefined radiological criterion to ensure internal consistency. In addition, all included cases required osteotomy and odontosection, ensuring a homogeneous surgical technique. Exclusion criteria included the presence of preoperative inferior alveolar nerve sensory disturbance, missing data regarding PRP exposure or primary outcome assessment, any systemic medical condition potentially affecting peripheral nerve function, wound healing, or tissue regeneration, and regular pharmacological treatment that could interfere with nerve healing, inflammatory response, or platelet function. Consequently, this cohort represents a selected high-risk population, and the observed incidence of neurosensory impairment is not intended to be extrapolated to unselected mandibular third molar extractions. 

In the PRP group, autologous PRP was obtained by venous blood extraction using four 9-mL dry tubes, followed by centrifugation at 2,500 rpm for 12 minutes. The PRP clot was manually separated and placed into the extraction socket after tooth removal (Figure 1) and wound closure was performed using resorbable 4/0 Vicryl sutures. In the non-PRP group, sockets were closed using the same suture material without PRP application, following standard clinical practice. To reduce confounding by indication, the cohort was restricted to a radiologically homogeneous high-risk population. 

*Collection of clinical data and endpoints*

Postoperative neurosensory assessment was performed as part of routine clinical follow-up and documented in the medical records. All patients underwent a standardized neurosensory evaluation at 1 week postoperatively. The primary endpoint was the presence of IAN neurosensory changes at the first postoperative follow-up 7 days after surgery. Neurosensory impairment was defined as any new postoperative hypoesthesia, anaesthesia, or dysesthesia reported by the patient in the inferior alveolar nerve sensory territory and documented in the clinical record. Recovery of neurosensory function was defined as restoration to at least 90% of the contralateral healthy side. This threshold was estimated by comparing the two-point discrimination distance measured on the operated side with that obtained on the contralateral unaffected side, which served as the individual reference value. When the discrimination threshold on the operated side approached that of the healthy side with minimal difference, corresponding to approximately 90% of the contralateral value, neurosensory function was considered functionally recovered. This approach allowed normalization of sensory measurements while accounting for inter-individual variability.

Secondary endpoints included time to complete neurosensory recovery, defined as the number of days from surgery to the first visit at which complete recovery was documented, and persistent neurosensory impairment, defined as the presence of sensory deficit at 6 months (a commonly accepted clinical threshold for persistent inferior alveolar nerve injury). Patients without neurosensory impairment at the 1-week visit did not undergo further dedicated neurosensory follow-up. Patients presenting with neurosensory impairment were followed monthly until complete recovery or until 6 months postoperatively. Patients who achieved recovery were discharged from follow-up.

*Statistical Analysis*

Continuous variables are presented as mean±standard deviation and categorical variables as counts and percentages. Baseline comparability between PRP and non-PRP groups was assessed using standardized mean differences. The incidence of inferior alveolar nerve impairment at 1 week was compared using absolute risk differences and odds ratios with 95% confidence intervals. Multivariable logistic regression adjusted for age and gender was used for the primary outcome. Among patients with impairment at 1 week, time to recovery was analysed using Kaplan-Meier methods and the log-rank test, with Cox regression used to explore factors associated with recovery. Persistence at final follow-up was analysed descriptively, with exploratory regression performed given limited power. Sensitivity analyses were conducted using inverse probability of treatment weighting. Statistical significance was set at p<0.05.

An a priori sample size estimation was performed for the primary endpoint based on proportions derived from previous studies of high-risk mandibular third molar extraction. Assuming an incidence of inferior alveolar nerve neurosensory impairment of 25% in the control group and an expected reduction to 12% in the PRP group, with a two-sided alpha level of 0.05 and 80% power, the required sample size was approximately 278 cases in total. Therefore, the final cohort of 300 extractions was considered adequate for the primary analysis.

## Results

*Study population*

A total of 300 patients were included in the analysis, of whom 144 received PRP and 156 did not. The mean age of the overall cohort was 33.7±15.5 years, and 67.3% of patients were female. Tooth laterality was evenly distributed, with 51.7% of extractions involving tooth 38 and 48.3% involving tooth 48. 

Patients in the PRP group were older than those in the non-PRP group (37.2±17.6 vs 30.5±12.5 years), corresponding to a moderate standardized mean difference (SMD=0.44). In contrast, gender distribution and tooth laterality were well balanced between groups, with both variables showing SMD values of approximately 0.09, indicating good baseline comparability for these covariates (Table 1).

*Primary outcome: Inferior alveolar nerve impairment at 1 week*

Inferior alveolar nerve impairment at 1 week was observed in 54 of 300 patients (18.0%). The incidence was higher in the non-PRP group (38/156, 24.4%) compared with the PRP group (16/144, 11.1%). The use of PRP was associated with an absolute risk reduction of 13.2% (95% CI -21.7 to -4.8), corresponding to a number needed to treat of 8 to prevent one case of IAN hypoesthesia at 1 week. The unadjusted odds ratio for IAN impairment associated with PRP was 0.39 (95% CI 0.21-0.73; p=0.004). In multivariable logistic regression analysis adjusting for age, gender, and tooth side, PRP use remained independently associated with a lower risk of IAN impairment (adjusted OR≈0.34; p=0.002). Age showed a non-significant trend toward increased risk, while gender and tooth laterality were not independently associated with the outcome. While age was not independently associated with injury incidence after adjustment, age-stratified analyses and predicted probability curves suggest a clinically relevant age-related trend. PRP use consistently reduced the predicted risk across all age ranges (Figure 2). 

*Time to recovery among patients with IAN impairment*

Among the 54 patients who developed IAN impairment at 1 week, 31 recovered during follow-up, whereas 23 remained unrecovered at the end of the follow-up period. The median time to recovery in the overall impaired cohort was approximately 120 days. When stratified by treatment group, the median time to recovery was 65 days in the non-PRP group, whereas the median was not reached in the PRP group within the follow-up period. Kaplan-Meier survival curves did not differ significantly between groups (log-rank p=0.22) (Figure 3). 

In Cox proportional hazards regression analysis, PRP use was not significantly associated with time to recovery (HR≈0.85; p=0.71). Increasing age was independently associated with slower recovery, with a hazard ratio of approximately 0.96 per additional year of age (p=0.016). When examining the proportion of patients with ongoing IAN impairment over time, between-group differences were evident at 1 week and 1 month, but were no longer statistically significant from approximately 2 months onward (p=0.103).

*Persistent inferior alveolar nerve impairment at final follow-up*

Persistent neurosensory impairment at final follow-up was observed in 14 of 38 patients (36.8%) in the non-PRP group and 9 of 16 patients (56.3%) in the PRP group. This difference was not statistically significant (OR≈2.20; 95% CI 0.67-7.23; p=0.24). In exploratory analyses adjusted for age, a trend toward a higher likelihood of persistence in the non-PRP group was observed (Figure 4).

*Sensitivity analysis: Propensity score weighting*

Inverse probability of treatment weighting achieved excellent balance across baseline covariates, with all post-weighting standardized mean differences below 0.02. In the IPTW-weighted analysis, PRP use remained significantly associated with a lower risk of IAN impairment at 1 week (OR≈0.35; p=0.001), confirming the robustness of the primary findings.

### Losses to Follow-Up

Five patients did not attend the scheduled postoperative review visit at 1 week following surgery and, consequently, their neurosensory status could not be assessed. As a result, these patients could not be classified into either the IAN impairment or non-impairment groups and were excluded from analyses. Importantly, no losses to follow-up occurred among patients who developed inferior alveolar nerve hypoesthesia**.**

## Discussion

The present study evaluated the effect of intraoperative platelet-rich plasma (PRP) on inferior alveolar nerve (IAN) neurosensory impairment following mandibular third molar extraction in a high-risk population. Our results suggest that PRP may be associated with a significant reduction in the incidence of IAN impairment at 1 week; however, these differences progressively diminished during follow-up, resulting in no clinically relevant differences in long-term neurosensory outcomes between PRP and non-PRP groups. Given that platelet-rich plasma is a safe autologous intervention with minimal associated risk, widely used in oral and maxillofacial practice and easily prepared in routine outpatient settings, it could represent a reproducible and cost-effective adjunct with potential clinical value.

*Early reduction in IAN impairment and anti-inflammatory effects of PRP*

Our findings further suggest that the clinical effect of PRP is most relevant during the acute postoperative phase. Differences between PRP and non-PRP groups were evident at 1 week and 1 month but were no longer statistically significant from approximately 2 months onward. From a clinical standpoint, the observed effect of platelet-rich plasma translates into a number needed to treat of 8, meaning that treatment of eight high-risk patients would prevent one case of early inferior alveolar nerve neurosensory impairment. Given the frequency of mandibular third molar surgery and the potential burden of postoperative neurosensory complications, the use of PRP could represent a clinically meaningful preventive benefit for these patients. Nevertheless, the clinical relevance of this finding should be interpreted in the context of the transient nature of most neurosensory disturbances and the absence of differences in long-term outcomes.

PRP is an autologous concentrate of platelets enriched in bioactive mediators released from platelet alpha granules after activation. These include platelet-derived growth factor (PDGF), transforming growth factor-β (TGF-β), vascular endothelial growth factor (VEGF), insulin-like growth factor-1 (IGF-1), and basic fibroblast growth factor (bFGF), all of which play a role in early tissue repair and modulation of inflammation [11]. Beyond their regenerative properties, platelets and PRP have demonstrated anti-inflammatory effects, including downregulation of proinflammatory cytokines such as interleukin-1β and tumor necrosis factor-α, and upregulation of mediators involved in resolution of inflammation, such as lipoxin A4 [11,12].

In the context of mandibular third molar surgery, particularly in cases requiring osteotomy and odontosection with intimate canal contact, acute postoperative inflammation and edema may contribute to transient nerve dysfunction through compression, ischemia, or neuroinflammatory mechanisms. By attenuating this early inflammatory response within the alveolus and surrounding tissues, PRP may reduce the likelihood that subclinical nerve irritation progresses to clinically detectable hypoesthesia.

Importantly, experimental and clinical studies suggest that the biological activity of PRP is most pronounced during the first days after application, with progressive decline as growth factors are released and degraded over time [11,13]. This temporal profile provides a biologically plausible explanation for our results: PRP could reduce early nerve impairment, but its effect appears to diminish as the acute inflammatory phase resolves and intrinsic healing mechanisms become dominant in both groups around two months after surgery.

*Neurotrophic and neuroregenerative potential of PRP*

In addition to its anti-inflammatory properties, our initial hypothesis was grounded in experimental and translational studies describing the presence of neuroprotective as well as neuroregenerative mediators within platelet-rich plasma. Platelets have been shown to release neurotrophic factors, including nerve growth factor (NGF) and brain-derived neurotrophic factor (BDNF), as well as IGF-1 and PDGF, which are involved in neuronal survival, neurite outgrowth, Schwann cell activation, angiogenesis, and remyelination [14-16]. Experimental models of peripheral nerve injury have demonstrated that PRP and PRP-derived matrices can create a microenvironment favourable to nerve repair [14,15]. Although direct clinical evidence in inferior alveolar nerve injury remains limited, these findings support the biological plausibility that PRP may protect neural structures in the immediate postoperative period and potentially facilitate early recovery of nerve function.

However, despite this biological rationale, our clinical findings indicate that PRP did not improve neurosensory outcomes once inferior alveolar nerve hypoesthesia was clinically established. These results suggest that, although PRP contains neurotrophic factors, these mechanisms may be insufficient to reverse established nerve dysfunction. Accordingly, PRP appears to act primarily as a preventive or early modulatory intervention rather than as a therapeutic strategy capable of improving long-term neurosensory outcomes once hypoesthesia has occurred. However, further randomized controlled studies are required to draw more definitive conclusions.

*Recovery patterns*

Among patients who developed IAN impairment at 1 week, PRP use was not associated with faster recovery or a lower risk of persistent impairment at final follow-up. At first glance, Kaplan-Meier curves suggested that recovery events occurred more frequently in the non-PRP group during later follow-up. Our findings suggest that PRP may shift recovery toward an earlier time course. Patients who benefit from PRP may recover before clinical hypoesthesia is established, preventing them from entering the cohort of patients with documented impairment at 1 week. Consequently, the remaining PRP patients with impairment at 1 week may represent a smaller and potentially more severe subgroup, while the non-PRP group includes a larger proportion of patients who recover later. To the authors’ knowledge, this phenomenon reflects differences in timing of recovery rather than overall regenerative capacity and may be influenced by conditioning analyses on a post-treatment outcome, with inherent risk of selection or collider bias.

Overall, long-term neurosensory outcomes tended to converge between groups, suggesting that PRP does not modify the final extent of nerve recovery once clinically evident injury has occurred. In this context, the higher proportion of hypoesthesia cases observed in the PRP group among patients with neurosensory impairment at 1 week should be interpreted with caution, as once again, this analysis conditions on a post-treatment outcome and is therefore susceptible to selection (collider) bias. It is plausible that PRP prevented a proportion of mild or transient hypoesthesia, resulting in a smaller and potentially more severe subset of impaired patients in the PRP group. Accordingly, when the entire cohort was considered and analyses were adjusted for age, PRP was not associated with an increased risk of persistent impairment and showed a trend toward a lower predicted probability of permanent hypoesthesia (Figure 2).

*Age imbalance and confounding by indication*

Baseline comparisons revealed that patients receiving PRP were older than those in the non-PRP group. Clinically, this is consistent with confounding by indication, in which surgeons may be more inclined-consciously or subconsciously-to use PRP in older patients perceived to be at higher risk of nerve injury and poorer recovery.

This imbalance is particularly relevant because increasing age has been consistently associated with delayed and incomplete recovery of IAN function (Figures 2 and 4). In a large multicentre retrospective cohort, Naruse *et al*. demonstrated that younger age was strongly associated with improved neurosensory recovery, whereas older age predicted poorer outcomes [10]. Importantly, this type of bias would be expected to act against demonstrating a protective effect of PRP. Therefore, the observation of a significant reduction in early IAN impairment in the PRP group-despite this unfavourable age distribution-strengthens the plausibility of a true beneficial association.

*Current alternatives*

Evidence regarding conservative or biological interventions for inferior alveolar nerve neurosensory impairment remains limited and heterogeneous. In a large multicentre retrospective cohort study, Naruse *et al*. evaluated factors associated with recovery from IAN neurosensory changes after mandibular third molar extraction and assessed commonly used conservative treatments, including corticosteroids and vitamin B12 supplementation [10]. Although postoperative corticosteroid use appeared to be associated with improved recovery in univariate analyses, this effect was attenuated after propensity score matching, highlighting both the potential relevance of early anti-inflammatory modulation and the challenges inherent to observational research in this field.

Beyond third molar surgery, the potential role of platelet-derived concentrates in nerve-related outcomes has been explored in the context of orthognathic surgery**,** particularly following bilateral sagittal split osteotomy [17], mandibular fractures [18] and other interventions such as lateralization of IAN [19]. In this setting, small clinical series and experimental studies have suggested that platelet-rich plasma or related autologous platelet concentrates may contribute to improving neurosensory outcomes. However, these studies are characterized by heterogeneous designs, limited sample sizes, variable outcome measures, and short follow-ups. Importantly, none has provided consistent evidence that platelet-derived concentrates can modify long-term neurosensory outcomes once clinically established nerve injury is present.

To our knowledge, there are currently no published clinical studies specifically evaluating the effect of platelet-rich plasma on inferior alveolar nerve outcomes following mandibular third molar extraction. In this context, our results suggest that PRP may be associated with a reduction in early neurosensory impairment, while not substantially influencing long-term recovery once hypoesthesia has become established. These findings help contextualize the biological rationale for PRP and support the interpretation that its potential benefit is more closely related to early neuroprotective and anti-inflammatory effects than to modification of the long-term course of established nerve injury.

*Limitations*

This study has several limitations. Its observational cohort design precludes definitive causal inference, and the conclusions are inherently less robust than those derived from randomized controlled trials. Although multivariable adjustment and propensity score analyses were used to mitigate confounding, residual bias cannot be fully excluded. 

PRP application was not randomized and may have been influenced by clinical judgment, resulting in confounding by indication, particularly related to patient age and perceived risk of nerve injury. While this bias would be expected to attenuate rather than exaggerate the observed protective association, it remains a limitation. In addition, this study was not designed to evaluate other factors that may modulate the effect of PRP, including differences in PRP preparation protocols, surgical variables (such as operative time, surgeon experience, or intraoperative difficulty), or patient-specific risk factors. Although all procedures followed the standard surgical approach used in our department for high-risk mandibular third molar extraction, the potential influence of these factors cannot be completely excluded. Further prospective randomized studies are needed to confirm these findings, better define the magnitude and duration of PRP effects, and identify patient subgroups most likely to benefit.

## Conclusions

In this study conducted in a high-risk population undergoing mandibular third molar extraction, intraoperative use of platelet-rich plasma was associated with a significant reduction in inferior alveolar nerve neurosensory impairment at one week. This early protective effect did not translate into differences in long-term neurosensory outcomes, suggesting that PRP primarily acts during the acute postoperative phase, likely through anti-inflammatory and neuroprotective mechanisms. Given its favourable safety profile, accessibility, and low cost, PRP may represent a useful preventive adjunct in selected high-risk cases. However, prospective randomized controlled trials are needed to confirm these findings and to better define its role in clinical practice.

## Figures and Tables

**Table 1:** Baseline demographic and clinical characteristics of the study cohort.

**Characteristic**	**Overall ** **(n=300)**	**PRP ** **(n=144)**	**No PRP ** **(n=156)**	**SMD**
**Age, mean±SD (yrs)**	33.7±15.5	37.2±17.6	30.5±12.5	0.44
**Female gender, n (%)**	202 (67.3)	100 (69.4)	102 (65.4)	0.09
**Tooth side 38, n (%)**	155 (51.7)	72 (50.0)	83 (53.2)	0.09
**Tooth side 48, n (%)**	145 (48.3)	72 (50.0)	73 (46.8)	0.09

SMD: Standardized mean difference. Values <0.10 indicate good balance between groups.

**Figure 1:**
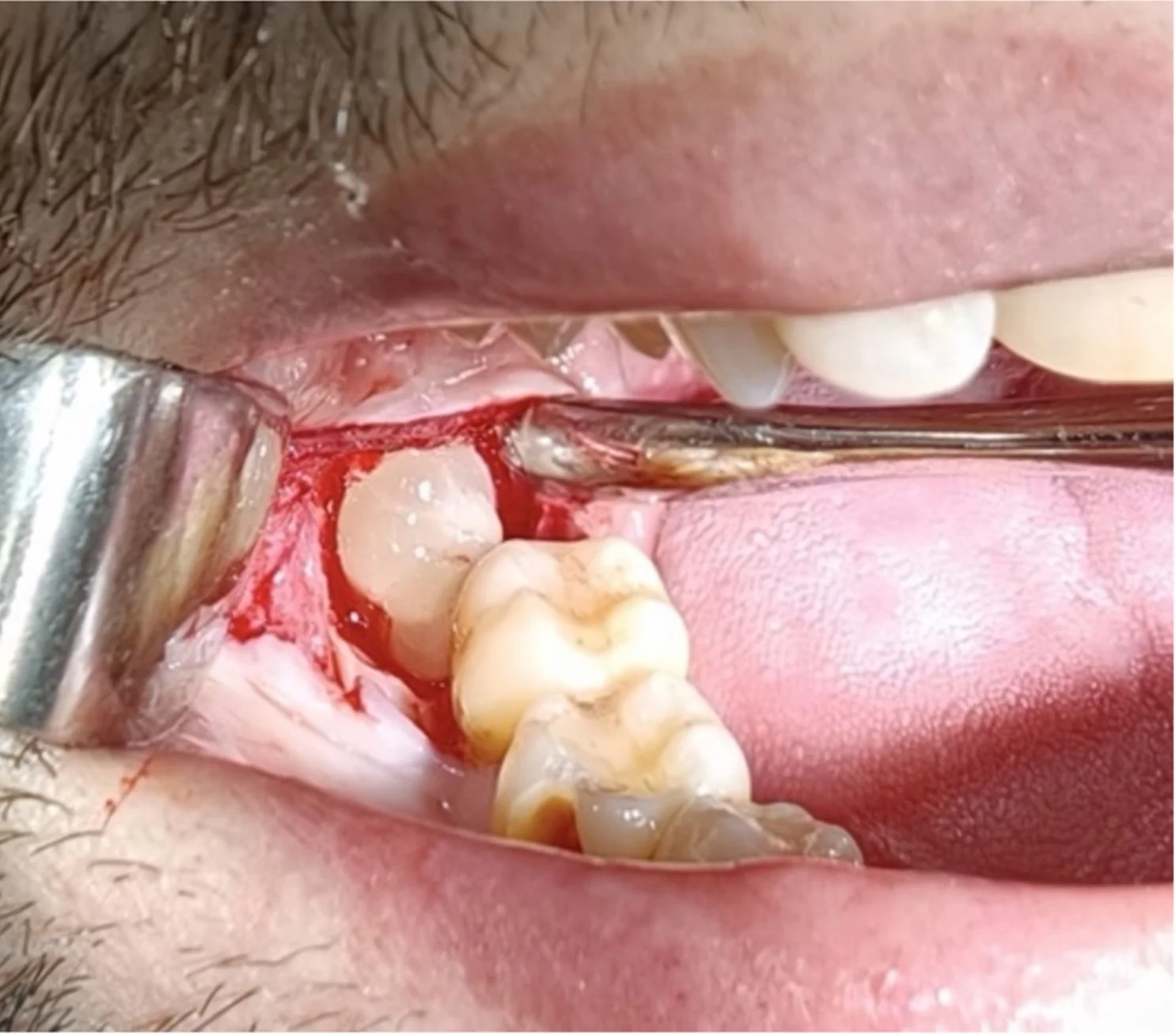
Fig. 1: PRP placement post extraction.

**Figure 2:**
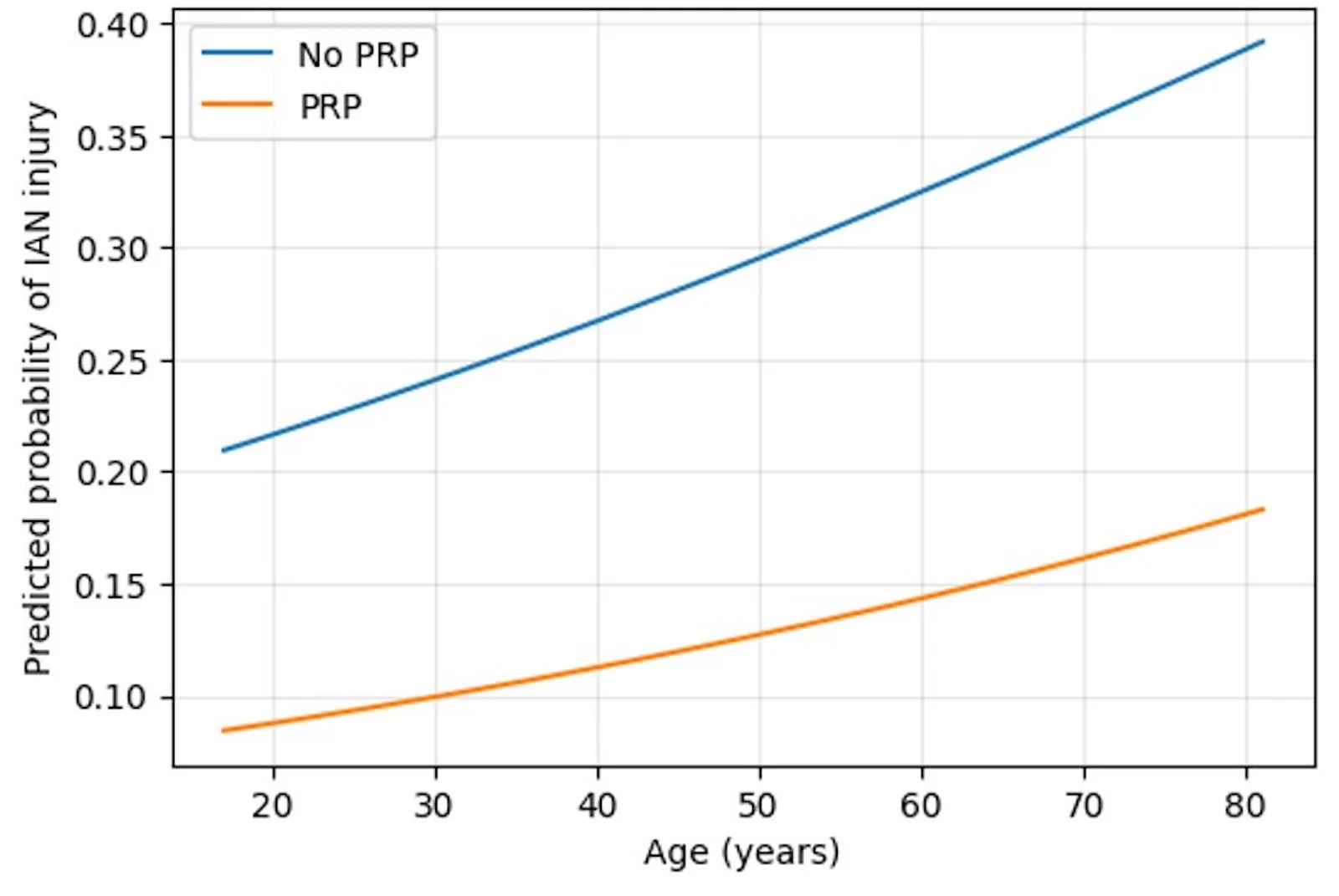
Fig. 2: Shows the predicted probability of inferior alveolar nerve injury at 1 week according to age, stratified by PRP use. An age-related increase in the predicted risk of nerve injury was observed in both groups. Across the entire age range, PRP use was consistently associated with a lower predicted probability of injury compared with no PRP.

**Figure 3:**
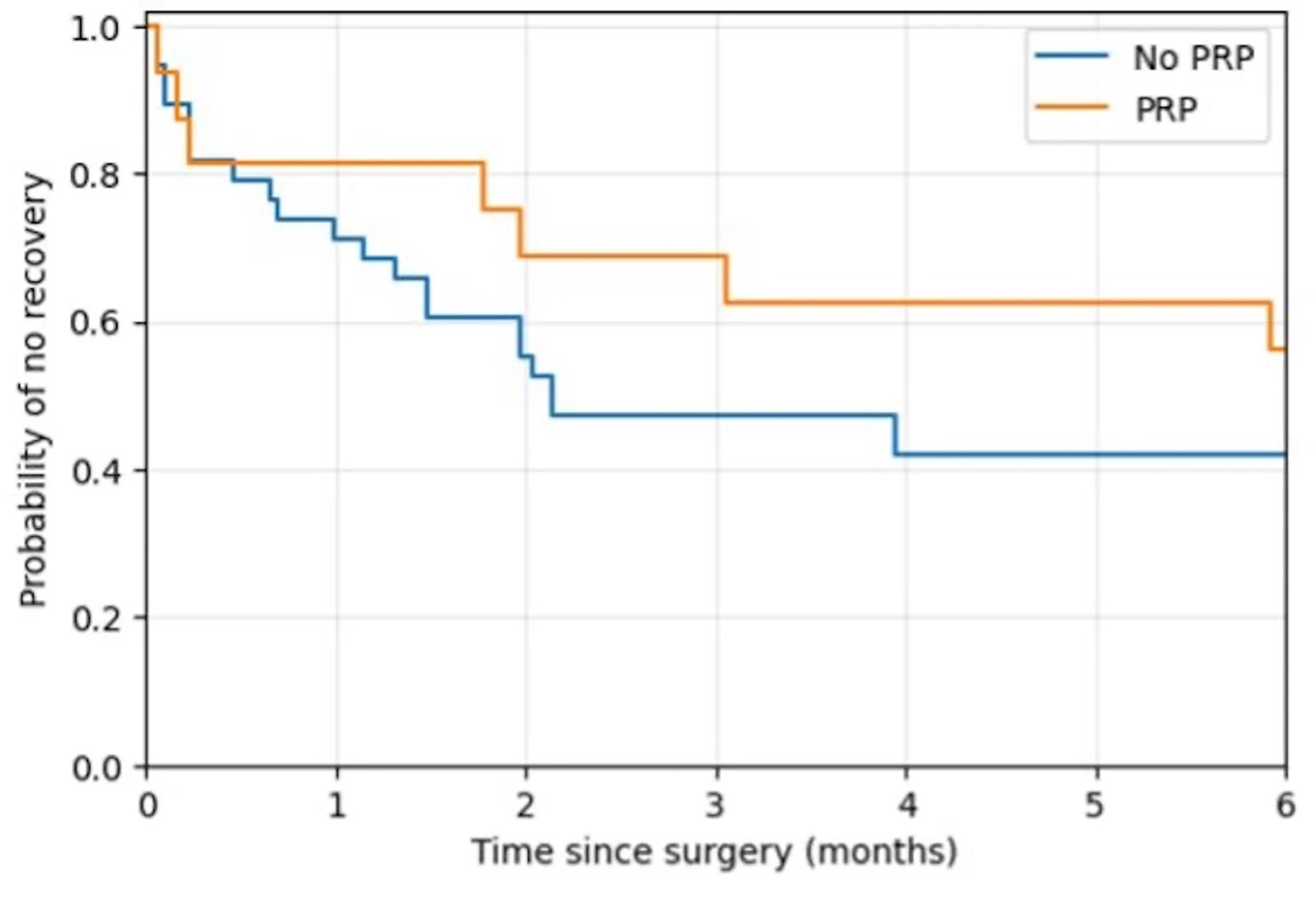
Fig. 3: Kaplan-Meier curves showing time to complete neurosensory recovery among patients with inferior alveolar nerve impairment at 1 week, stratified by PRP use. No significant difference between groups was observed (log-rank p=0.22).

**Figure 4:**
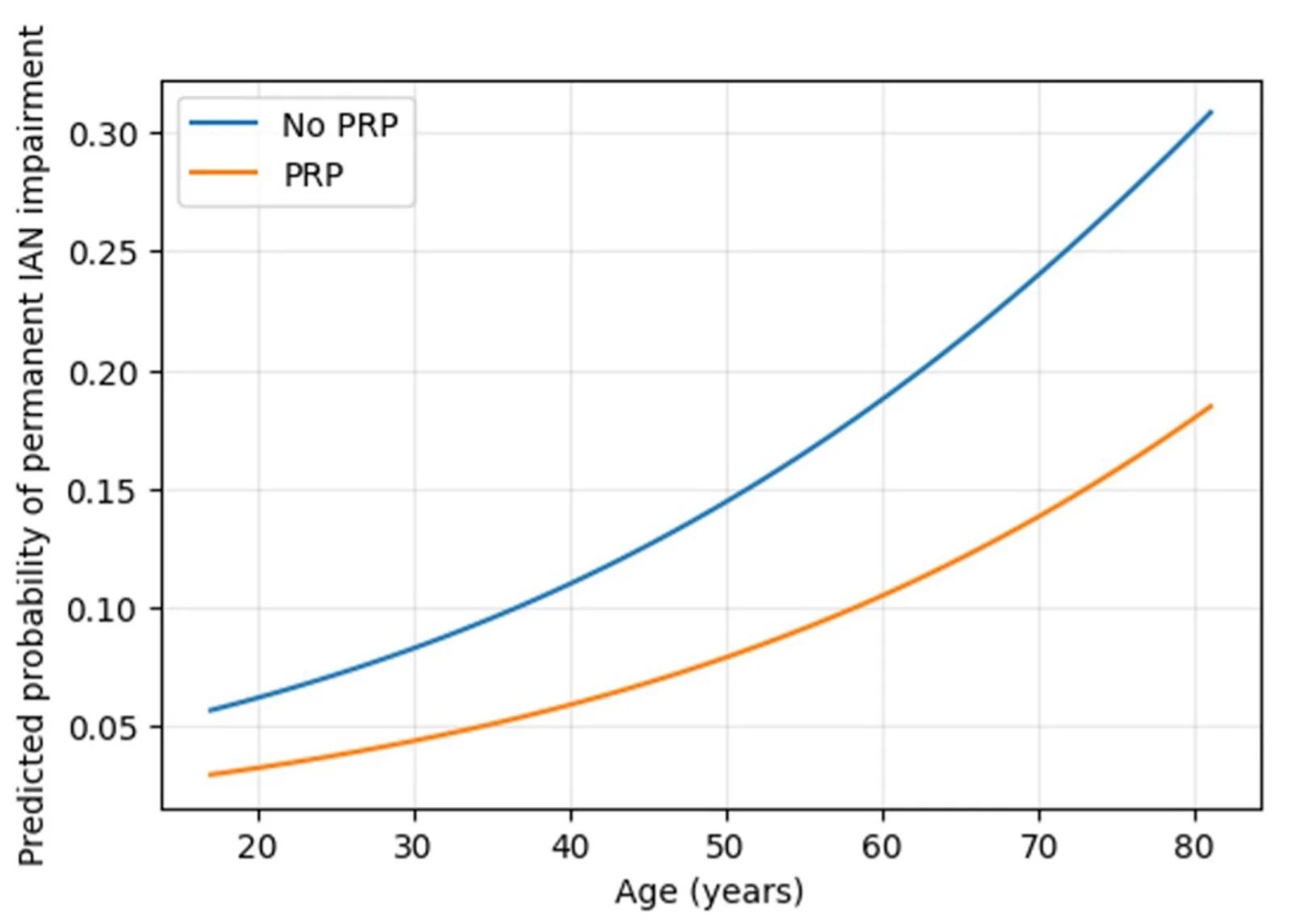
Fig. 4: Predicted probability of permanent inferior alveolar nerve impairment at 6 months by age stratified by PRP use. Predicted probabilities were derived from a logistic regression model including age and PRP use. Increasing age was associated with a higher predicted probability of persistent neurosensory impairment, while PRP use showed a consistently lower predicted probability across the age range, although this difference did not reach statistical significance.

## Data Availability

The data that support the findings of this study are available from the corresponding author upon reasonable request.

## References

[1] Patel PS, Shah JS, Dudhia BB, Butala PB, Jani YV, Macwan RS (2020). Comparison of panoramic radiograph and cone beam computed tomography findings for impacted mandibular third molar root and inferior alveolar nerve canal relation. Indian J Dent Res.

[2] Del Lhano NC, Ribeiro RA, Martins CC, Assis NMSP, Devito KL. Panoramic versus CBCT used to reduce inferior alveolar nerve paresthesia after third molar extractions: A systematic review and meta-analysis. Dentomaxillofac Radiol. 2020;49:20190265. 10.1259/dmfr.20190265 PMC7213531 31724883

[3] AlAli AM, AlAnzi TH (2021). Inferior alveolar nerve damage secondary to orthodontic treatment: A systematic scoping review. Int J Risk Saf Med.

[4] Coulthard P, Kushnerev E, Yates JM, Walsh T, Patel N, Bailey E, Renton TF. Interventions for iatrogenic inferior alveolar and lingual nerve injury. Cochrane Database Syst Rev. 2014;(4):CD005293. 10.1002/14651858.CD005293.pub2 PMC10794896 24740534

[5] McArdle LW, Patel N, Jones J, McDonald F (2018). The mesially impacted mandibular third molar: The incidence and consequences of distal cervical caries in the mandibular second molar. Surgeon.

[6] Bailey E, Kashbour W, Shah N, Worthington HV, Renton TF, Coulthard P. Surgical techniques for the removal of mandibular wisdom teeth. Cochrane Database Syst Rev. 2020;(7):CD004345. 10.1002/14651858.CD004345.pub3 PMC7389870 32712962

[7] Albanese A, Licata ME, Polizzi B, Campisi G (2013). Platelet-rich plasma (PRP) in dental and oral surgery: From wound healing to bone regeneration. Immun Ageing.

[8] Bao M, Du G, Zhang Y, Ma P, Cao Y, Li C (2021). Application of platelet-rich fibrin derivatives for mandibular third molar extraction-related postoperative sequelae: A systematic review and network meta-analysis. J Oral Maxillofac Surg.

[9] Ye L, He Y, Ma W, Zhou F, Liu J (2024). Effect of platelet-rich fibrin on recovery after third molar surgery: A systematic review and meta-analysis. J Craniomaxillofac Surg.

[10] Naruse T, Suyama K, Fukushima H, Nakamura N, Yamashita K, Furukawa K, Morishita K, Rokutanda S, Yamada T (2025). Recovery from inferior alveolar neurosensory changes after lower third molar extraction: A multicenter, retrospective study. Medicine (Baltimore).

[11] El-Sharkawy H, Kantarci A, Deady J, Dyke TEV (2007). Platelet-rich plasma: Growth factors and pro- and anti-inflammatory properties. J Periodontol.

[12] Xu P, Wu Y, Zhou X, Yang Z, Zhang X, Hu X, Yang J, Wang M, Wang B, Luo G, He W, Cheng B (2020). Platelet-rich plasma accelerates wound healing by modulating inflammation and angiogenesis. Int J Mol Sci.

[13] Marx RE (2004). Platelet-rich plasma: Evidence to support its use. J Oral Maxillofac Surg.

[14] Etulain J, Mena HA, Meiss RP, Frechtel G, Gutt S, Negrotto S, Schattner M (2018). An optimised protocol for platelet-rich plasma preparation to improve its angiogenic and regenerative properties. Sci Rep.

[15] Zhang H, Liu X, Wu J (2020). PRP-based biomaterials for peripheral nerve repair. Biomater Sci.

[16] Sánchez M, Anitua E, Delgado D (2017). Platelet-rich plasma, a source of neurotrophic factors. Curr Pharm Biotechnol.

[17] Nasiri M, Ghannadpour A, Hakimiha N (2025). Efficacy of blood products and photobiomodulation therapy for neurosensory recovery in patients with inferior alveolar nerve injury following orthognathic surgery: A systematic review study. J Lasers Med Sci.

[18] Tabrizi R, Moslemi H, Shafiei S, Dastgir R, Peacock ZS. Does platelet-rich fibrin enhance recovery from neurosensory disturbance following mandibular fractures? A double-blind, split-mouth randomized clinical trial. Craniomaxillofac Trauma Reconstr. 2024;17(4):NP90-NP96. 10.1177/19433875241257737 PMC11562979 39553808

[19] Khojasteh A, Hosseinpour S, Nazeman P, Dehghan MM (2016). The effect of a platelet-rich fibrin conduit on neurosensory recovery following inferior alveolar nerve lateralization: A preliminary clinical study. Int J Oral Maxillofac Surg.

